# Glucagon-Like Peptide-1 Induced Signaling and Insulin Secretion Do Not Drive Fuel and Energy Metabolism in Primary Rodent Pancreatic β-Cells

**DOI:** 10.1371/journal.pone.0006221

**Published:** 2009-07-13

**Authors:** Marie-Line Peyot, Joshua P. Gray, Julien Lamontagne, Peter J. S. Smith, George G. Holz, S. R. Murthy Madiraju, Marc Prentki, Emma Heart

**Affiliations:** 1 Molecular Nutrition Unit and Montreal Diabetes Research Center at the Centre de Recherche du Centre Hospitalier de l'Université de Montréal and Departments of Nutrition and Biochemistry, Université de Montréal, Montréal, Quebec, Canada; 2 BioCurrents Research Center (NIH:NCRR), Marine Biological Laboratory, Woods Hole, Massachusetts, United States of America; 3 Department of Chemistry, United States Coast Guard Academy, New London, Connecticut, United States of America; 4 State University of New York, Upstate Medical University, Syracuse, New York, United States of America; University of Bremen, Germany

## Abstract

**Background:**

Glucagon like peptide-1 (GLP-1) and its analogue exendin-4 (Ex-4) enhance glucose stimulated insulin secretion (GSIS) and activate various signaling pathways in pancreatic β-cells, in particular cAMP, Ca^2+^ and protein kinase-B (PKB/Akt). In many cells these signals activate intermediary metabolism. However, it is not clear whether the acute amplification of GSIS by GLP-1 involves in part metabolic alterations and the production of metabolic coupling factors.

**Methodology/Prinicipal Findings:**

GLP-1 or Ex-4 at high glucose caused release (∼20%) of the total rat islet insulin content over 1 h. While both GLP-1 and Ex-4 markedly potentiated GSIS in isolated rat and mouse islets, neither had an effect on β-cell fuel and energy metabolism over a 5 min to 3 h time period. GLP-1 activated PKB without changing glucose usage and oxidation, fatty acid oxidation, lipolysis or esterification into various lipids in rat islets. Ex-4 caused a rise in [Ca^2+^]_i_ and cAMP but did not enhance energy utilization, as neither oxygen consumption nor mitochondrial ATP levels were altered.

**Conclusions/Significance:**

The results indicate that GLP-1 barely affects β-cell intermediary metabolism and that metabolic signaling does not significantly contribute to GLP-1 potentiation of GSIS. The data also indicate that insulin secretion is a minor energy consuming process in the β-cell, and that the β-cell is different from most cell types in that its metabolic activation appears to be primarily governed by a “push” (fuel substrate driven) process, rather than a “pull” mechanism secondary to enhanced insulin release as well as to Ca^2+^, cAMP and PKB signaling.

## Introduction

The mechanisms of glucose-stimulated insulin secretion (GSIS) in the β-cell remain to be defined. In addition to the triggering pathway involving a rise in ATP production, K_ATP_ channel closure and a Ca^2+^ rise [1], [2], [3], fuel signaling is thought to involve additional pathways, in particular anaplerosis/cataplerosis, pyruvate cycling processes, endogenous lipolysis and enhanced glycerolipid/fatty acid (GL/FFA) cycling [4], [5], [6], [7], [8], [9]. Besides the signals induced by calorigenic nutrients and their associated production of metabolic coupling factors [4], β-cell function is modulated by a variety of neurohormonal agents and glucoincretins [10], including glucagon like peptide-1 (GLP-1), an incretin hormone secreted by the L-cells of the distal intestine [10], [11]. GLP-1 levels in the plasma increase rapidly following a meal [12], and this hormone has a profound glucose-lowering effect through both central and peripheral actions [13], the latter effect being particularly at the level of the β-cell [10]. GLP-1 stimulates insulin gene expression [14], proinsulin biosynthesis [10], and it also potentiates GSIS [10], [14]. GLP-1 also has proliferative [15] and antiapoptotic actions on the β-cell [10]. The biologically active form of GLP-1 is derived from proglucagon via the action of prohormone convertase enzymes [10], [11], and circulating GLP-1 is rapidly removed from the circulation following its degradation by dipeptidyl peptidase-4 (DPP-4) [16].

GLP-1 exerts its cellular action by binding to its receptor, a G-protein coupled receptor (GLP-1R), expressed in β-cells, nervous system, heart and kidney [10], [11]. The activation of the GLP-1R leads to the induction of many signal transduction systems, including cAMP, Ca^2+^, PI3-Kinase and EGF receptor signaling [10], [11], [17], [18]. These multiple actions of GLP-1 are also observed upon exposure of β cells to Exendin-4 (Ex-4), a peptide that is an incretin mimetic and which lowers levels of blood glucose as a consequence of its ability to activate the GLP-1R [10]. GLP-1 induces insulin secretion during short-term exposure to the hormone, or after chronic exposure to the hormone [10], [19]. Even though the precise mechanisms of GLP-1 action are not fully understood, it is established that the stimulation of GSIS by GLP-1 involves activation of membrane-bound adenylyl cyclase and cAMP production, leading to protein kinase-A (PKA) and Epac [20] activation, and an increase in intracellular Ca^2+^ [10], [11], [21]. A rise in cytoplasmic and mitochondrial Ca^2+^ has been linked to the activation of mitochondrial dehydrogenases, in particular pyruvate dehydrogenase [22], α-ketoglutarate dehydrogenase and isocitrate dehydrogenase [3], [22], [23]. Additionally, islet tissue and the β-cell contain some glycogen [24], [25] that might be mobilized following a rise in cellular Ca^2+^ or cAMP [26], thus releasing glucose-1P that may enter the glycolytic pathway following its conversion to glucose-6-P. It is therefore attractive to hypothesize that GLP-1 may indirectly activate β-cell energy metabolism, thereby raising levels of cellular ATP, and possibly influencing other metabolic coupling factors, via its effect on cellular levels of Ca^2+^ and cAMP. Thus, it is generally believed that the cAMP and Ca^2+^ pathways cannot fully account for the complete magnitude of GLP-1 mediated GSIS enhancement [10], [11], [19].

Besides Ca^2+^ signaling, the binding of GLP-1 to its receptor also results in the activation of protein kinase-B (PKB/Akt) [27], [28], [29] and PKB activation in other cell types has been linked to various metabolic effects, including glucose transport in muscle [30] glycogen synthesis [31] and lipolysis [32]. It can also be hypothesized that an increase in cellular ATP content might inactivate AMP-activated protein kinase (AMPK) [33], as elevated glucose does [34], which may lead to reduced phosphorylation of hormone sensitive lipase and adipose triglyceride lipase [35], with subsequent enhanced lipolysis [36] and activation of the lipid amplification arm of glucose signaling for insulin secretion [36]. Thus, our previous work has established a role for lipolysis in GSIS [37], [38]. In addition, it was previously shown that orlistat, a pan-inhibitor of lipases, suppresses the incretin action of GLP-1 [39], and that GLP-1 enhances lipolysis in HIT (β) cells [40].

In the present study we examined whether the acute stimulation of GSIS by GLP-1 or Ex-4 involves the modulation of glucose, fatty acid and energy metabolism in the β-cells, as studied using isolated islets of both rats and mice. The study was designed to respond to four questions of general interest for β-cell neuropeptide and fuel signaling. 1) Does GLP-1 amplify GSIS in part by metabolic signaling? 2) Does the activation of major cellular signaling processes (Ca^2+^, cAMP, PKB etc) in response to a physiological peptide agonist changes β-cell metabolism? 3) Does enhanced insulin secretion contribute significantly to total energy consuming processes in the β-cell? 4) Is the β-cell similar or different from most tissues in term of metabolic activation, specifically whether it is primarily governed by a “push” (fuel substrate driven) process, rather than a “pull” mechanism secondary to enhanced activation of its major cellular function.

## Materials and Methods

### Animals and diets

#### Ethics Statement

All procedures were performed in accordance with the Institutional Committee for the Protection of Animals at the Centre Hospitalier de l'Université de Montréal or Institutional Guidelines for Animal Care (IACUC) at the Marine Biological Laboratory, in compliance with United States Public Health Service regulations.

Wistar rats (200–250 g; Charles River) and male Swiss-Webster mice or male CD-1 rats were housed under controlled temperature (21°C) and light conditions (12-h light/dark cycle) with free access to water and standard chow diet.

### Isolation and culture of islets and islet cells

Pancreatic islets were isolated by collagenase digestion of the pancreas according to Gotoh et al [41]. After digestion and washing and separation by histopaque gradient centrifugation, islets were hand-picked and cultured overnight in a humidified incubator with 5% CO_2_. For measurements of insulin secretion, the cell culture medium was RPMI-1640 containing 11 mM glucose and supplemented with 10% foetal calf serum, 10 mM HEPES (pH 7.4), 1 mM sodium pyruvate, 100 U/ml penicillin and 100 µg/ml streptomycin (RPMI complete medium). For measurements of oxygen consumption, isolated rat or mouse islets were cultured overnight in RPMI-1640 complete medium containing 5 mM glucose. For single cell studies of mitochondrial ATP levels, the islets were dispersed by incubation in Ca^2+^/Mg^2+^ free phosphate buffered saline, 3 mM EGTA and 0.002% trypsin as previously described [42]. Islet cells obtained by dispersion of islets were plated on poly-D-lysine coated coverslips (MatTek, Ashland, MA) in 35 mm Petri dishes. After 24 h, single islet cells were transduced with Ad-MitoLuc-RFP at 50 MOI (multiplicity of infection) for 12 h, after which viral media were replaced with appropriate growth media. Transduction efficiency in single islet cells, determined from RFP fluorescence, reached more then 90% under these conditions.

### Measurement of insulin secretion and insulin content

After overnight culture, rat islets were distributed in 12-well plates (10 islets/well) and incubated for 2 h in 1 ml RPMI complete medium containing 2.8 mM glucose. The islets were then washed and pre-incubated for 45 min at 37°C in KRBH/0.07% defatted BSA and 2.8 mM glucose, followed by incubation for 1 h in 1 ml KRBH/0.5% defatted BSA and 2.8, 8.3 or 16.7 mM glucose plus or minus 20 nM GLP-1-(7–36)-amide (Bachem Americas, Torrance, CA, USA) or 20 nM Ex-4 (Bachem Americas, Torrance, CA, USA), in the presence or absence of 0.3 mM palmitate. For whole mouse islets, or populations of single mouse islet cells (plated on the wells of 48-well plates), insulin secretion was measured at 4, 7.5 and 16.7 mM glucose in the presence or absence of 10 nM Ex-4. At the end of a 30 min static incubation, media were kept for insulin measurement by radioimmunoassay (Linco research, St. Charles, MO, USA). Islet total insulin content was measured following acid-ethanol (0.2 mM HCl in 75% ethanol) extraction.

### Islet fatty acid oxidation and esterification

Fatty acid (FA) oxidation and esterification were determined in batches of 50 islets cultured as described above. After 2 h incubation in 2.8 mM glucose-RPMI complete medium, islets were washed in KRBH/0.25% BSA and pre-incubated for 45 min at 37°C in KRBH/0.25% defatted BSA and 2.8 mM glucose after which they were incubated for 2 h (FA oxidation) or 4 h (FA esterification) in 1 ml KRBH/0.25% defatted BSA containing 2.8, 8.3 or 16.7 mM glucose in the presence or absence of 20 nM GLP-1, 0.1 mM (oxidation) or 0.2 mM (esterification) palmitate, 1 µCi/ml [9,10(n)-^3^H] palmitate (51 Ci/mmol, GE Healthcare, Baie d'Urfé, QC, Canada), and 1 mM carnitine. At the end of the incubation, the media were collected for the determination of islet FA oxidation and total lipids were extracted from islets for the measurement of islet FA esterification [38].

### Glucose oxidation and utilization

Groups of 20 islets cultured and pre-incubated as described above for the insulin secretion assay, were incubated in a 0.6 ml Eppendorf tube without capping, in a final volume of 70 µl KRBH/0.25% defatted BSA containing 2.8 to 16.7 mM glucose with D-[U-^14^C]-glucose for oxidation measurements (250 mCi/mmol, PerkinElmer, Canada) and D-[5-^3^H]-glucose for utilization measurements (16 Ci/mmol, GE Healthcare, Canada) with or without 20 nM GLP-1 [43]. For oxidation incubations, this incubation tube was placed upright in an airtight-sealed 20 ml scintillation vial, which also contained an empty 1.5 ml Eppendorf tube without capping. The reaction was stopped after 90 min incubation at 37°C with constant agitation by the addition of 50 µl of a mix consisting of metabolic poisons (400 mM citric acid, 10 µM rotenone, 10 µM antimycin and 3.5 mg KCN, pH 4.9). To the empty 1.5 ml tube in the scintillation vial, 250 µl of 5% (w/v) KOH was added to trap released ^14^CO_2_. Incubations were continued for 60 min at room temperature and glucose oxidation was determined by measuring the KOH-trapped ^14^CO_2_. For utilization measurements, the scintillation vial also contained 500 µl of 1 mM HCl at the bottom. After stopping the incubations as above, the tightly sealed vials were left at room temperature for 40 h and glucose utilization was determined by measuring the amount of ^3^H_2_O equilibrated into the 0.5 ml HCl in the vial.

### Lipolysis

Batches of 60 islets, cultured as described above, were washed in KRBH/0.07% BSA and 2.8 mM glucose and were transferred into 0.2 mL of KRBH/0.07% BSA medium in a 48 well plate with 2.8, 8.3 or 16.7 mM glucose and 20 nM GLP-1 or 20 nM Ex-4. The plate was incubated for 3 h at 37°C in a humidified atmosphere containing 5% CO_2_, after which the media were collected for glycerol determination by an enzymatic assay [37]. Islet protein content was measured, as previously described [37].

### Measurement of [Ca^2+^]_i_ and cAMP content

Mouse islets were dispersed by mild digestion with trypsin-EDTA and the single islet cells were plated on glass cover-slips for [Ca^2+^]_i_ measurement, or in 96-well cell culture plates for cAMP determination. After overnight culture, the islet cells were infected with Ad-MtLuc-RFP (m.o.i. equal to 50). Measurements of [Ca^2+^]_i_ were performed after 48 h, using fura-2 loaded β-cells, imaged at 100X magnification using a dual excitation light source and a ratiometric imaging system (IonOptix Corp.) equipped with filter sets that minimize crossover between fura-2 and RFP [44]. The cAMP content was determined by immunoassay using a Direct Biotrak EIA kit (Amersham) as described earlier [45].

### Oxygen Consumption

Oxygen consumption in single rat or mouse islets was measured at 37°C in the presence or absence of 10 nM Ex-4 or 10 µM forskolin by the self-referencing method based on an electrochemical oxygen sensor (BioCurrents Center, MBL, Woods Hole, MA) moving between a “near” and “far” position at the islet. The magnitude of the amperometric current used for the reduction of oxygen is proportional to the oxygen concentration at that particular point [46]. When islet respires, oxygen concentration is lower at near position. Thus, the current used for reduction of oxygen on the sensor will be greater at the far position, and the measured difference in the electric current between far and near position (Difference Current, DC) is greater than zero. When an islet further increases oxygen consumption (in response to a rise in glucose concentration), oxygen concentration at near position decreases even more and causes further increase in the DC. Oxygen consumption was measured in islets incubated in KRBH containing 4, 7.5, and 16.6 mM glucose.

### Mitochondrial ATP

Changes of mitochondrial ATP levels (ATPm) were measured in a population of approximately 250,000 single rat or mouse islet cells infected with Ad-MitoLuc-RFP. This virus was generated using the mt-Luc coding sequence in plasmid VR102. mt-Luc is a fusion protein in which the 26 amino acid N-terminal signal peptide of cytochrome C oxidase subunit VIII (COX8) is fused to codon-optimized firefly luciferase [47]. Ad-MitoLuc-RFP infected islet cells were incubated with KRBH buffer (for 72 h) containing, either 4, 7.5 or 16.6 mM glucose without or with 10 nM Ex-4. Single islet cells, grown and infected on the PDL-coated glass coverslips inside a 35 mm dish (MatTek, Ashland, MA) were placed directly onto the surface of the photocathode optical window of a Hamamatsu R464 photomultiplier tube housed in a 37°C heated box. Luciferin was then added to the KRBH at a final concentration of 100 µM in order to allow the measurement of photoemissions resulting from luciferase-catalyzed oxidation of luciferin [48].

### PKB/Akt phosphorylation

A group of 200 islets cultured and pre-incubated as described for insulin secretion experiments, was incubated for 30 min in 1 ml KRBH/0.5% defatted BSA containing 2.8 or 8.3 mM glucose in the presence or absence of 20 nM GLP-1. After 30 min, islets were lysed in 0.1 ml of 50 mM HEPES (pH 7.5), 2 mM sodium orthovanadate, 4 mM EDTA, 100 mM sodium fluoride, 10 mM sodium pyrophosphate, 1 mM PMSF, 1% (v/v) NP40 and protease inhibitors. Total cellular proteins were obtained after sonicating the islets for 10 s and centrifugation at 15,000×g at 4°C for 12 min. The supernatant was collected and the protein content assayed (Pierce). Proteins were resolved by 10% sodium dodecyl sulfate-polyacrylamide gel electrophoresis (SDS-PAGE) and electrotransferred to nitrocellulose membranes (BioRad, Hercules, CA, USA). After overnight blocking with 5% non-fat milk, the membranes were probed with antibodies for pSer^473^-PKB and total PKB (Cell Signaling Technology, MA, USA) and the proteins were visualized by enhanced chemiluminescence (Pierce).

### Statistical analysis

Data are expressed as means±SE. Significance was calculated for multiple comparisons by using one-way analysis of variance (ANOVA) with Bonferroni post-hoc testing. A *P*-value of<0.05 was considered significant.

## Results

**Figure 1:**
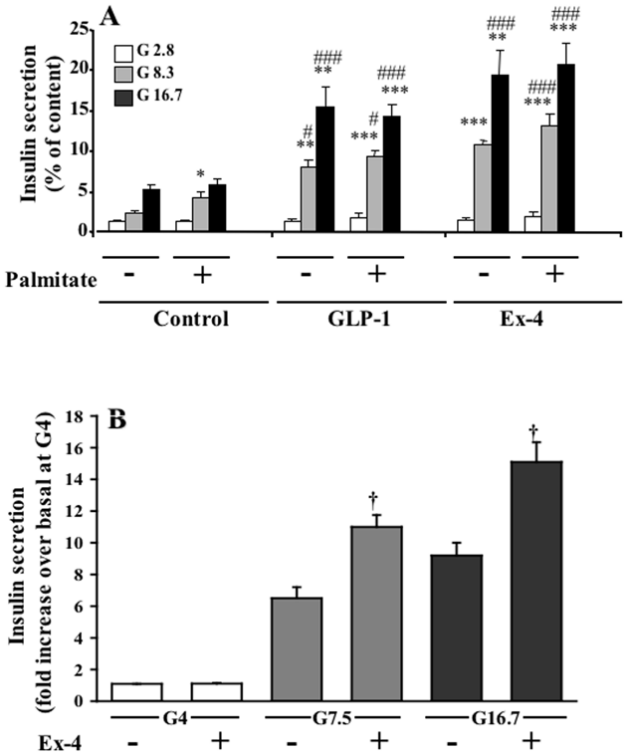
Acute effects of GLP-1 and Ex-4 on GSIS in rat (A) and mouse (B) islets. Pancreatic islets were isolated and cultured overnight prior to use as described in Methods. Islets were incubated for 1 h (A) or 30 min (B) as described in Methods for examining insulin secretion at indicated concentrations of glucose and GLP-1 (20 nM) or Ex-4 (20 nM) in A, or 10 nM Ex-4 in B, in the absence or presence of 0.3 mM palmitate. Insulin released into the media and the total islet insulin content were measured. Results shown are mean±SE from 3 independent experiments with quadruplicates (n = 12). For A, *p<0.05, **p<0.01, ***p<0.001 when compared with corresponding 2.8 mM glucose group. #p<0.05, ##p<0.01, ###p<0.001 when compared with corresponding groups without GLP-1 or Ex-4 treatment. For B, †p<0.01 when compared to corresponding minus Ex-4 group. Insulin secretion in mouse islets at basal glucose levels (4 mM) was 1.56±0.3 ng insulin/10 islets/30 min.

Glucose stimulated insulin secretion from isolated rat islets in a dose-dependent manner, and this effect was enhanced by 0.3 mM palmitate at an intermediate concentration (8.3 mM) of glucose (Fig. 1A). Both GLP-1 and Ex-4 markedly potentiated GSIS at 8.3 and 16.7 mM glucose, and palmitate did not further elevate insulin secretion. At 16.7 mM glucose, GLP-1 and Ex-4 stimulated insulin secretion 3–4 fold more than glucose alone. In the presence of GLP-1 or Ex-4 the amount of insulin released during the 45 min time period corresponded to approximately 20% of the total islet content of insulin. In mouse islets, Ex-4 enhanced GSIS at 7.5 and 16.7 mM glucose, but not at 4 mM glucose (Fig. 1B). Overall, Fig. 1 shows that the islets that were used in the current study were highly responsive to glucose, GLP-1 and Ex-4 and therefore suitable to be used to respond to the addressed questions. Inasmuch as both GLP-1 and Ex-4 are agonists of GLP-1 receptor on the β-cells, with near equal potency, most studies to date have not found any significant differences between these two agonists in *in vitro* experiments. In the present study, although most experiments were performed with both GLP-1 and Ex-4, because of the similar nature of the data, only either of these is illustrated.

**Figure 2:**
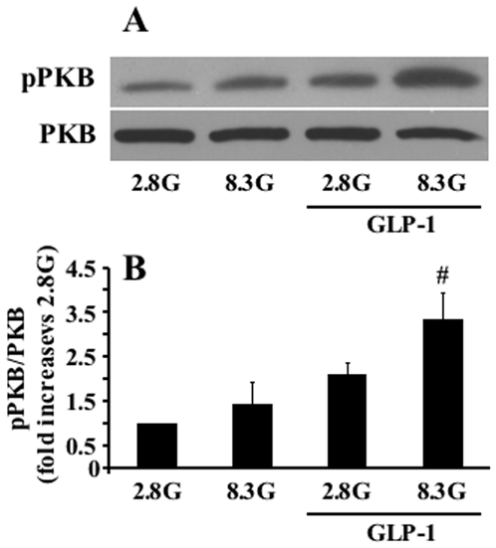
PKB phosphorylation in response to GLP-1 in rat islets. Islets were incubated for 30 min at 2.8 or 8.3 mM glucose in the presence or absence of 20 nM GLP-1. Activation of PKB by GLP-1 was assessed with antibodies specific for Ser^473^phospho-PKB and PKB, respectively. (A) Representative immunoblot of phospho- and total PKB in rat islets. (B) Quantitative measurement of PKB phosphorylation after 30 min treatment with GLP-1. Results are means±SE of 4 separate experiments. #p<0.05, when compared to the corresponding ‘minus GLP-1’ group.

PKB has multiple metabolic effects in various cell types [49] and GLP-1 acutely activates PKB in INS cells [27] and human islets [29]. So far it has not been shown that GLP-1 activates PKB in normal rodent β-cells. We examined the effect of GLP-1 (20 nM) on PKB phosphorylation in rat islets after a 30 min exposure to the hormone, and observed that GLP-1 significantly increased Ser-473 phosphorylation of PKB in normal islet cells (Fig. 2).

**Figure 3:**
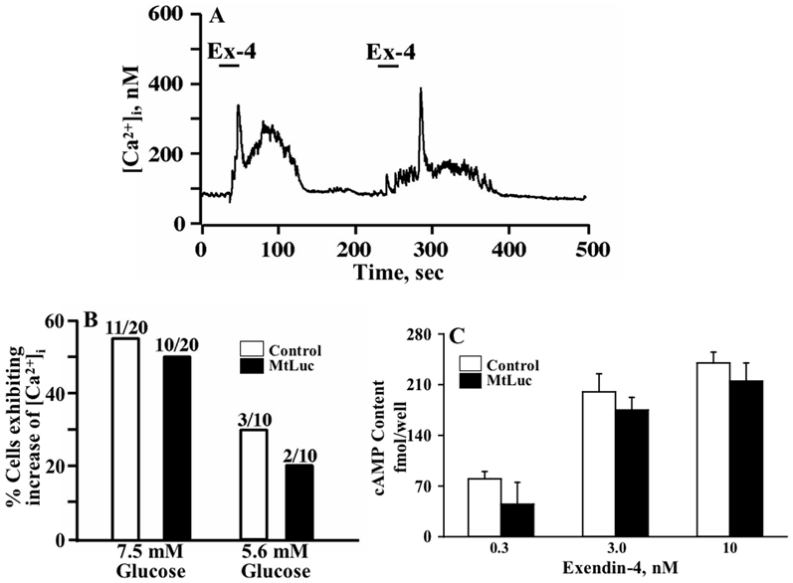
Exendin-4 increases [Ca^2+^]_i_ and cAMP content in mouse β-cells. A, A single fura-2 loaded and Ad-MtLuc-RFP-infected mouse β-cell was imaged to determine the [Ca^2+^]_I,_ at 5.6 mM glucose in KRBH. After establishment of a stable baseline [Ca^2+^]_i_, 10 nM Ex-4 was applied for 25 sec (indicated by horizontal bars). Note that a repeatable increase of [Ca^2+^]_i_ was measured. B, Population study conducted at the single cell level in which the action of Ex-4 to increase [Ca^2+^]_i_ was evaluated in β-cells not infected (open bars) or infected with Ad-MtLuc-RFP (filled bars). For these experiments, the KRB contained 5.6 or 7.5 mM glucose, as indicated. A response to Ex-4 was defined as a >100 nM increase of [Ca^2+^]_i_ occurring in a single β-cell. C, Ex-4 caused a dose-dependent increase in cAMP content in mouse islet cells in KRB containing 7.5 mM glucose without or with Ad-MtLuc-RFP infection.

It was therefore of interest to determine whether changes in islet intermediate metabolism might be explained by influences of GLP-1 on PKB phosphorylation/activation [50], [51], in addition to its previously demonstrated stimulatory effects on islet cAMP and Ca^2+^ signaling shown in many studies employing both normal (human, rat, mouse) and tumoral β-cell (for reviews see [3], [10], [19], [20], [21]). Tsuboi and co-workers [52] reported that GLP-1 receptor activation increased [Ca^2+^]_i_, which caused an elevation of mitochondrial ATP in MIN6 insulin-secreting cells. In order to ascertain that Ex-4 increases [Ca^2+^]_i_ and cAMP in normal mouse β-cells under our experimental conditions where mitochondrial ATP and O_2_ consumption were measured, single cell measurements of [Ca^2+^]_i_ were performed using β-cells loaded with fura-2, infected with Ad-MtLuc-RFP, and equilibrated in KRB containing 5.6 mM glucose. Under these conditions, Ex-4 (10 nM) stimulated an increase of [Ca^2+^]_i_ in these cells (Fig. 3A). Importantly, MtLuc expression had no effect on the percentage of cells exhibiting a >100 nM increase of [Ca^2+^]_i_ (Fig. 3B). Thus, viral infection did not disrupt the stimulatory action of Ex-4 on intracellular Ca^2+^ signaling. Since Ex-4 is known to stimulate insulin secretion in a glucose-dependent manner, we examined whether glucose concentration influences Ex-4 stimulated intracellular Ca^2+^ signaling. This was in fact the case since the action of Ex-4 to increase [Ca^2+^]_i_ was more prominent under conditions in which mouse β-cells were equilibrated in KRB containing 7.5 mM glucose as compared to 5.6 mM glucose (Fig. 3B).

Since it is known that the increase of [Ca^2+^]_i_ in response to Ex-4 is secondary to β-cell cAMP production [44], it was of interest to determine if viral infection altered the ability of Ex-4 to increase levels of cAMP in primary mouse islet cells. We found that Ex-4 stimulated cAMP production in a dose-dependent manner, in islet cells infected with Ad-MtLuc-RFP (Fig. 3C), without any significant difference from cells not infected with Ad-MtLuc-RFP (Fig. 3C). Thus, viral infection as described here for Ad-MtLuc-RFP, did not disrupt intracellular Ca^2+^ signaling and cAMP production in mouse β-cells.

**Figure 4:**
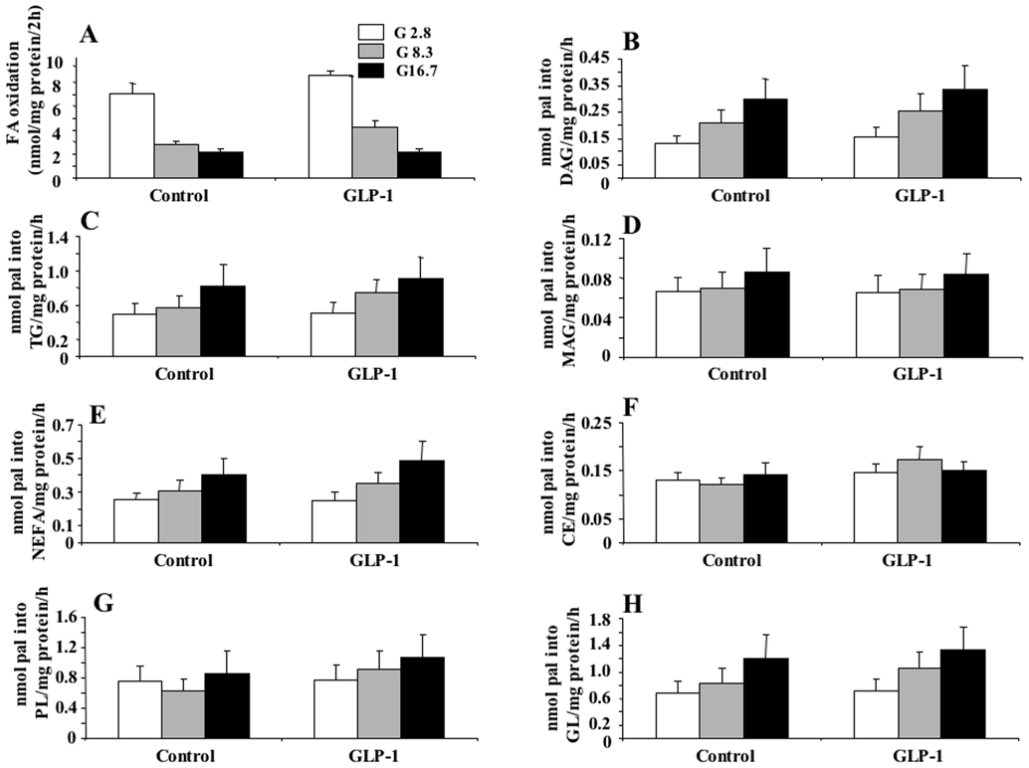
Palmitate β-oxidation and esterification into different lipids in rat islets in the absence or presence of GLP-1. Islets were processed as described for insulin secretion (see [Fig. 1]) and after the pre-incubation step they were incubated for 2 h (FA oxidation) or 4 h (FA esterification) in 1 ml KRBH/0.25% defatted BSA containing medium with 1 mM carnitine and 1 µCi/ml [9,10(n)-^3^H] palmitate (51 Ci/mmol), at 2.8, 8.3 or 16.7 mM glucose in the presence or absence of 20 nM GLP-1. Cold palmitate (pal) was present at 0.1 mM for oxidation and 0.2 mM for esterification experiments. A, Palmitate oxidation; B—H, palmitate incorporation into diacylglycerol, DAG (B), triacylglycerol, TG (C), monoacylglycerol, MAG (D), non-esterified fatty acids, NEFA (E), cholesterol esters, CE (F), phospholipids, PL (G) and total glycerolipids, GL (H). Means±SE of 6–8 separate incubations in 3 independent experiments.

We have previously shown [53], [54] that GSIS in rat islets and INS cells is accompanied by reduced β-oxidation and increased partitioning of fatty acids into glycerolipids, an event that is thought to be coupled to β-cell activation of insulin release [5]. Therefore, we examined whether the acute stimulatory effect of GLP-1 on GSIS in islets might result from altered lipid metabolism. There was no significant effect of GLP-1 on palmitate oxidation or its incorporation into different classes of glycerolipids or cholesterol esters or phospholipids (Fig. 4) at the various tested glucose concentrations.

**Figure 5:**
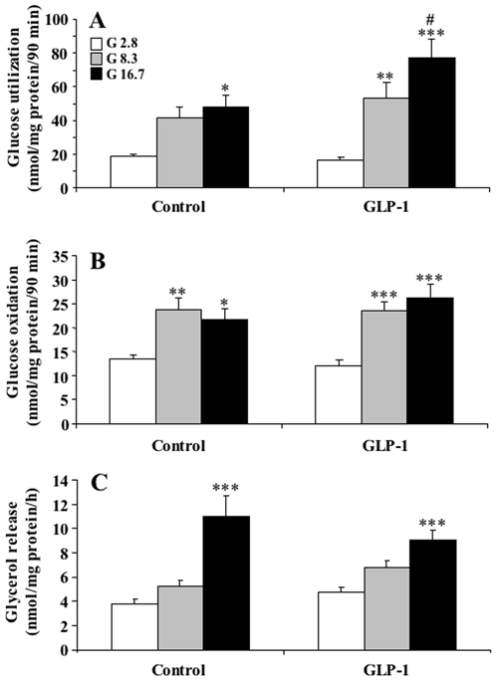
Glucose metabolism and lipolysis in rat islets in the absence or presence of GLP-1. Islets were processed as described for insulin secretion (see [Fig. 1]) and after the pre-incubation step they were incubated in 70 µl KRBH/0.25% defatted BSA medium containing 2.8, 8.3 or 16.7 mM glucose plus or minus 20 nM GLP-1 in presence of D-[U-^14^C]-glucose (for oxidation) (A) and D-[5-^3^H]-glucose (for utilization) (B). Incubations were stopped after 90 min as described in Methods. Glucose oxidation was measured as ^14^CO_2_ released, and glucose utilization was determined by measuring the amount of released^ 3^H_2_O. Results are means±SE of 15 determinations in 3 separate experiments. *p<0.05, **p<0.01, ***p<0.001 when compared to the corresponding 2.8 mM glucose group; #p<0.05 when compared to the corresponding ‘minus GLP-1’ group. For lipolysis determinations (C) overnight-cultured rat islets were washed in KRBH/0.07% BSA medium with 2.8 mM glucose and were transferred into 0.2 mL KRBH/0.07% BSA medium with 2.8, 8.3 or 16.7 mM glucose with or without 20 nM GLP-1. After incubation for 3 h at 37°C, glycerol released into the media and the islet protein content were determined. Means±SE from 4 independent experiments with pentaplicates.

We next assessed whether the acute stimulatory effect of GLP-1 on GSIS is associated with changes in islet glucose metabolism. However, rat islet glucose utilization (Fig. 5A) or oxidation (Fig. 5B) was not significantly affected by GLP-1 at all tested glucose concentrations, except for a small increase in utilization at 16.7 mM glucose (Fig. 5A).

Enhanced lipolysis and GL/FFA cycling are thought to play a role in the amplification (K_ATP_-independent) arm of fuel induced insulin secretion [5], [35], and previous work in the HIT cell line showed that GLP-1 enhances glycerol release in this tumoral (β) cell [40]. However, GLP-1 did not enhance lipolysis in rat islets at low, intermediate and high glucose (Fig. 5C).

**Figure 6:**
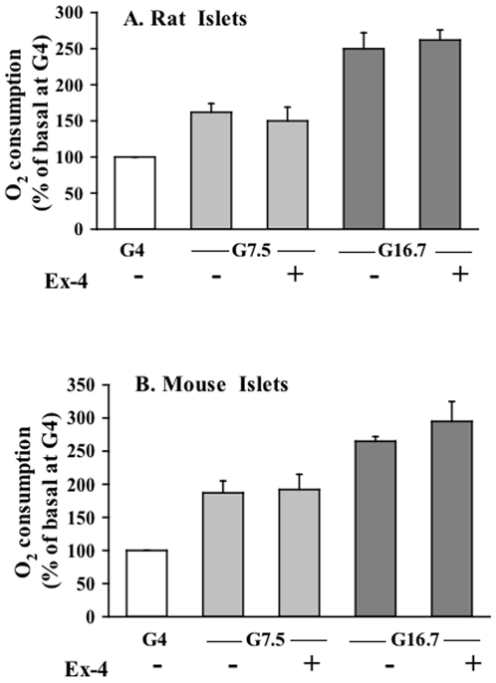
Oxygen consumption of rodent islets in the absence or presence of Ex-4. Single rat (A) and mouse (B) islets were adhered on glass coverslips inside a 35 mm dish using CellTak adhesive. After 30 min equilibration at 4 mM glucose, oxygen consumption was measured in response to 4, 7.5 and 16.6 mM glucose with and without 10 nM Ex-4 (10 nM). Data are means±SE of 3 experiments.

We also examined the effect of Ex-4 and GLP-1 on oxygen consumption at different glucose concentrations in rat and mouse islets since this parameter reflects overall fuel utilization and metabolic activation of a given tissue. Even though oxygen consumption increased with glucose concentration, there was no significant change with Ex-4 (Fig. 6A & B) or with GLP-1 (data not shown), in accordance with the results from glucose oxidation experiments. Also, the adenylate cyclase activator forskolin (10 µM) did not affect oxygen consumption in mouse islets (data not shown).

**Figure 7:**
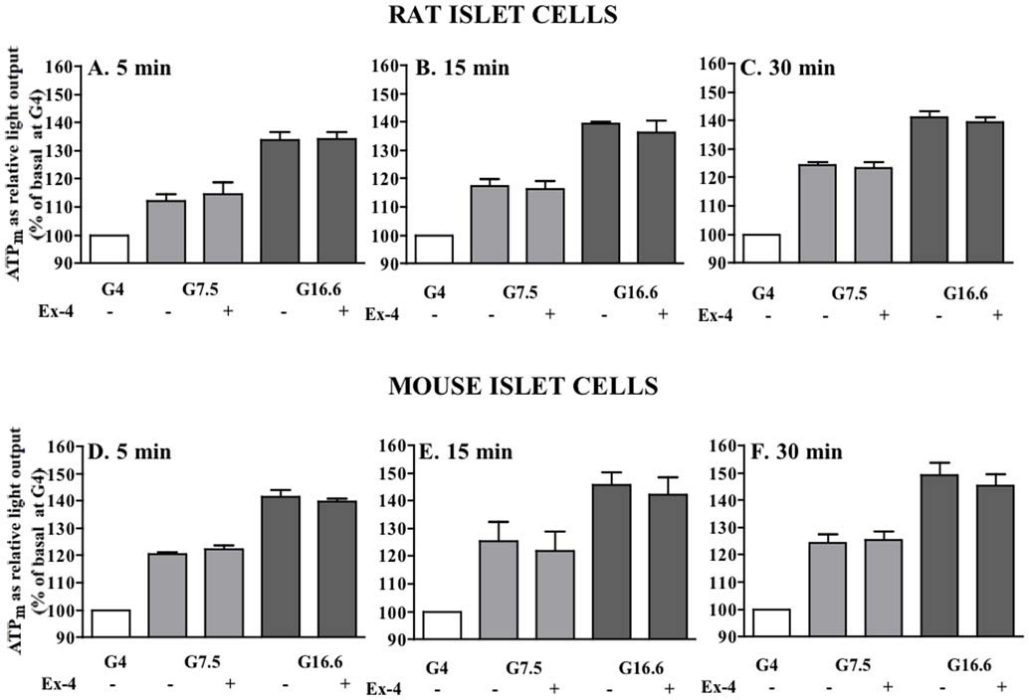
Mitochondrial ATP levels of rodent islet cells in the absence or presence of Ex-4. Dispersed rat (A,B,C) and mouse (D,E,F) islet cells were transduced with Ad-MtLuc-RFP. ATP levels in the presence of 4, 7.5 and 16.6 mM glucose with and without 10 nM Ex-4 were determined as photoemission resulting from luciferase-catalyzed oxidation of luciferin, in populations of approximately 250,000 single islet cells at 5, 15 and 30 min. Data are means±SE of 3 experiments.

Enhanced mitochondrial metabolism and ATP production plays a central role in β-cell fuel signalling [55] and GLP-1 was shown to enhance mitochondrial ATP production in the tumoral β-cell line MIN6 [52]. We further measured mitochondrial ATP levels in isolated rat and mouse islet cells engineered to express mitochondrial-targeted luciferase, after incubation for 5 to 30 min, with different glucose concentrations and Ex-4. Glucose caused a marked and dose dependent increase in mitochondrial ATP, within 5 min of incubation, in both rat and mouse islets cells, but Ex-4 did not significantly change mitochondrial ATP at all tested glucose concentrations (Fig. 7A & B). Identical results were obtained using 20 nM GLP-1 (data not shown). We verified that the mitochondrial-luciferase-engineered islet cells respond normally to the respiratory substrates and inhibitors, by examining the effect of methylsuccinate (10 mM), a membrane permeable form of succinate and FCCP (10 µM), an uncoupler of oxidative phosphorylation. As expected, during a 15 min incubation, methylsuccinate enhanced ATP production above the basal (4 mM glucose) level, whereas, FCCP reduced ATP levels (data not shown).

## Discussion

Fuel stimulated insulin secretion in the β-cells involves the production of metabolic coupling factors and an elevation in intracellular Ca^2+^ [1]. GLP-1 and Ex-4 enhance GSIS in the β-cells, cause a rise in cytosolic Ca^2+^, elevate cAMP and activate the PKA and PKB signaling pathways [3], [10], [19], [20], [21]. Because activation of Ca^2+^, cAMP and PKB signaling is known to modulate intermediary and energy metabolism in several cell types, we hypothesized that GLP-1 signaling to stimulate insulin secretion is in part linked to changes in β-cell metabolism and the production of metabolic coupling factors. The data in fact show that GLP-1 or Ex-4 barely affect β-cell metabolism at large, and therefore that GLP-1 induced insulin secretion may not involve metabolic signaling related to glucose, lipid and energy metabolism.

We [1], [5] and others [56] provided evidence that enhanced lipolysis in β-cells plays a role in GSIS. However, lipolysis does not appear to be involved in the acute amplification of GSIS by GLP-1 in normal islet tissue. Thus, in accordance with a previous study using isolated mouse islets [57], we observed that GLP-1 or Ex-4 do not affect rat islet lipolysis. Furthermore, islets from hormone sensitive lipase-KO mice exhibited GLP-1 stimulation of GSIS similar to that of control mouse islets [37]. The previously reported increase in lipolysis by acute treatment with GLP-1 in HIT (β) cells [40] is probably due to the inherent differences in established tumoral cell lines from normal islet β-cells.

Lipid amplification pathways of GSIS in the β-cell involve reduced fatty acid β-oxidation and concomitant increased esterification of fatty acids into glycerolipids [53], [54]. We have suggested that enhanced GL/FFA cycling is instrumental in the amplification of GSIS via the generation of lipid signalling molecules that act as metabolic coupling factors for insulin secretion [5], [35]. However, no effect of GLP-1 was noticed on either palmitate β-oxidation or its incorporation into glycerolipids, cholesterol esters or phospholipids. Thus, GLP-1 mediated enhancement of GSIS does not appear to be dependent on either lipolysis or alteration in GL/FFA cycling.

GLP-1 activates PKB in INS (β)-cells [27], [28], human [29] and rat islets (present study). PKB activation can lead to various metabolic effects in different cell types, including glucose transport [30] and glycogen synthesis [31] in muscle, and lipolysis in adipocytes [32]. In addition PKB activation inhibits AMPK [33], and activation of AMPK by AICAR or expression of a constitutively active AMPK mutant in β-cells curtail GSIS [34], [58]. However, despite GLP-1 activation of islet PKB, none of the studied parameters of β-cell metabolism were changed, suggesting that β-cell PKB activation is not linked to acute changes in β-cell metabolism.

Measurements of mitochondrial ATP, which rapidly increase within 5 min and respiration as oxygen consumption in rat and mouse islets revealed no change with Ex-4, in accordance with glucose oxidation measurements showing no effect of GLP-1. This indicates that GLP-1 does not amplify GSIS via changes in β-cell energy metabolism. Earlier work on the acute actions of GLP-1 on β-cell energy metabolism using the tumoral β-cell line MIN-6 documented that high concentrations (100 nM) of GLP-1 rapidly increased levels of ATP in both the cytosol and the mitochondrial matrix [52]. In contrast, we now report that activation of the GLP-1 receptor by 10 nM Ex-4 failed to alter levels of ATP in the mitochondrial matrix of both rat and mouse islet cells over a period of 5–30 min at different glucose concentrations. Even though in the present study and in [52], early changes in the mitochondrial ATP were measured (within few minutes of incubations), the different results obtained in [52] could be attributable to the use of excessively high GLP-1 concentration and also the tumoral cell line, MIN6. It is also possible that the tumoral β-cells appear to differ from normal β-cells as far as GLP-1 action on β-cell metabolism is concerned, both in terms of lipolysis and energy metabolism. It is possible that the interaction of the endoplasmic reticulum with the mitochondria, as described by Tsuboi and co-workers [52], occurs in MIN6 cells but not in normal rodent β−cells. Thus, Ca^2+^ released from the endoplasmic reticulum might not be a strong stimulus for mitochondrial ATP production in authentic β-cells.

Collectively the present results indicate that alterations of glucose, lipid and energy metabolism as well as ATP production are not involved in the mechanisms whereby GLP-1 augments GSIS. Perhaps of greater importance is the established ability of GLP-1 to stimulate cAMP production and to activate both PKA and Epac [19], [20]. These two cAMP-binding proteins regulate β-cell functions that are also under the control of glucose metabolism. Such functions include K_ATP_ channel activity, cytosolic Ca^2+^ handling, and insulin granule exocytosis. The present results also show that the higher glucose concentration-dependency of Ex-4 to stimulate insulin secretion is reflected in a similar high glucose concentration dependence for Ex-4-mediated [Ca^2+^]_i_ increase in mouse β-cells. Ex-4-stimulated cAMP production, which precedes [Ca^2+^]_i_ increase in islet cells [21], is not influenced by Ad-MtLuc-RFP viral infection indicating that the viral infection did not adversely affect the results of this study. Thus, the insulin secretagogue action of GLP-1 likely arises as a consequence of its ability to facilitate the action of glucose-derived metabolic coupling factors, rather than by directly stimulating metabolic signaling per se.

Another aspect of the present study is that it provides new information about β-cell activation of energy metabolism of this fuel sensing cells with respect to other tissues. In many cell types like muscle tissue, the activation of their primary biological function and energy demanding process drives mitochondrial metabolism via changes in redox, phosphorylation potential or Ca^2+^. In the present study, it came as a surprise that in spite of a very significant enhancement in GSIS by GLP-1 and Ex-4 at high glucose in both rat and mouse islets, amounting to approximately 20% of the total insulin content in 1 h (in the case of rat islets), no concomitant rise in fuel utilization, O_2_ consumption or ATP production was noticed. These results indicate that the energy consuming processes activated by GLP-1 and likely other glucoincretins, such as ion ATP-ases, the release of insulin from the ready-releasable pool of secretory granules, the ATP-dependent [59] refilling of this pool and recycling of excess plasma membrane via endocytosis following exocytosis, consume very little energy relatively to overall cell metabolism.

Recent studies in fact revealed that much of the energy needed for the secretory granule membrane fusion comes from the conformational changes of the proteins involved in this process. Thus, the energy needed for the fusion of membranes overcoming the repulsive forces, arises from the formation and folding of v- and t-SNARES [60]. *In vitro* experiments using artificial lipid bilayers demonstrated that the formation of a single SNARE complex (the v- and t-SNARE complex) provides sufficient energy for the fusion of the outer leaflets of the bilayers [61]. Release and re-cycling of v- and t-SNARES in high-energy form is accomplished by the involvement of the SNAP and NSF ATPase, with the hydrolysis of ATP, which is the only step where metabolic energy is invested [60]. Other proteins including Munc18–1 are also known to provide additional conformational energy to facilitate the fusion of SNARE complexes [60]. Thus, it is likely that the acute augmentation of GSIS at high glucose by GLP-1 is not dependent on elevated substrate oxidation and ATP production but may utilize the Ca^2+^-mediated and SNAREpin/Munc protein conformational energy dependent fusion of the docked secretory granules with plasma membrane to release their insulin content. Therefore, it seems that the insulin secretion process *per se* does not consume much of the β-cell metabolic energy even upon marked insulin release.

Finally, the data indicate that the β-cell is different than most cell types in terms of energy metabolism where activation of Ca^2+^, cAMP, PKB etc signaling promoted by a stimulus drives simultaneously a biological process (for example contraction), and cellular metabolism/ATP production to support it. Thus, β-cell metabolic activation appears to be primarily driven by substrate (fuel) availability, a “push” process [1], [62] rather than a “pull” mechanism secondary to enhanced insulin release. However the data do not discount the possibility that a marked rise in Ca^2+^ influx promoted by a potent secretagogue like glucose drives mitochondrial metabolism [63]. The present study also emphasizes the major differences that exist between normal and tumoral β-cell in term of metabolic activation.
